# Modeling the critical causal factors of postharvest losses in the vegetable supply chain in eThekwini metropolitan municipality: The log-linear regression model

**DOI:** 10.1016/j.heliyon.2024.e39565

**Published:** 2024-10-18

**Authors:** Siphesihle Qange, Lelethu Mdoda, Asanda Mditshwa

**Affiliations:** a Discipline of Agricultural Economics, School of Agricultural, Earth and Environmental Sciences, University of KwaZulu Natal, P/Bag X01, Scottsville, Pietermaritzburg, 3209, South Africa; b Discipline of Horticultural Sciences, School of Agricultural, Earth and Environmental Sciences, College of Agriculture, Engineering and Science, University of KwaZulu Natal, P/Bag X01, Scottsville, Pietermaritzburg, 3209, South Africa

**Keywords:** Causal factors, Log-linear regression, Postharvest losses, Smallholder vegetable farmers, Vegetable supply chain

## Abstract

Vegetables, rich in essential bioactive compounds, are crucial for human health and vital to the global food system. However, the supply chain for vegetables is intricate, marked by product seasonality, demand variability, and limited shelf life. These factors contribute to significant losses, with 44 % of global vegetable production wasted at various stages in the food chain. Postharvest losses (PHLs) are a considerable issue, posing risks to food security and economic sustainability. Despite numerous interventions aimed at reducing PHLs, a comprehensive understanding of the primary causes remains insufficient, preventing the implementation of practical solutions. The study investigates the factors causing PHLs in the vegetable supply chain. A structured questionnaire was used to collect the data from 238 farmers. The study adopted a cross-sectional research design and a multi-stratified random sampling method. Descriptive statistics and log-linear regression were used to analyze the data. The results revealed that 56 % of the farmers were women, with an average age of 45 and a household size of five. Most farmers had completed 12 years of education, with 76 % being unemployed and 78 % depending on farming as their primary source of income. The regression analysis showed that age, distance to markets, and lack of transportation were significant factors at the 1 % level. In contrast, education, farming experience, market access, and weather conditions were significant at the 5 % level. The study recommends policies promoting innovative postharvest technologies alongside investment in infrastructure to mitigate these losses.

## 1 Introduction

Vegetables, rich in bioactive compounds essential for human health, significantly contribute to the global food supply, balanced nutrition, culinary variety, and cultural traditions [15,17,20,37,38]. Research indicates that vegetables help prevent chronic diseases such as cardiovascular diseases, diabetes, and certain cancers [40]. The vegetable supply chain is subject to considerable postharvest losses (PHLs), with 44 % of fresh vegetables wasted globally and 37 % in Sub-Saharan Africa, translating to 120–170 kg per capita annually [9]. In South Africa alone, poor post-harvest handling and value-addition techniques result in over 10 million tons of food waste [37].

The complexity of the vegetable supply chain, characterized by factors such as seasonality, demand fluctuations, and short shelf lives, poses significant challenges [1]. Proper handling and prompt processing are essential to minimize losses due to vegetables' perishability [35]. Despite technological advancements in packaging, cold chain logistics, and postharvest practices, their adoption remains limited in resource-constrained regions [20]. Vegetables' inherent perishability and global supply chain complexities exacerbate the risk of postharvest losses [22,36].

Postharvest losses happen between harvesting and consumption, primarily due to inadequate harvesting, handling, storage, and transportation practices [32,41[32,41]. Inadequate infrastructure and storage facilities further exacerbate these losses, particularly in developing countries [36]. Economically, postharvest losses impact producers, increase food prices, and undermine food security, nutrition, and economic development [3,39].

The environmental consequences of postharvest losses include resource depletion, greenhouse gas emissions, and environmental pollution [28]. Efforts to mitigate these losses face challenges such as limited access to appropriate technologies and infrastructure, especially in rural areas, and stakeholders' lack of awareness and training [22,36]. However, opportunities exist to reduce postharvest losses through advances in technology, infrastructure development, and capacity-building initiatives [14,26]. This study aims to identify and investigate the primary factors contributing to postharvest losses in the vegetable supply chain. By understanding these factors, the study seeks to provide actionable recommendations to stakeholders for developing effective interventions and strategies to reduce losses and enhance the sustainability of the vegetable supply chain.

## 2 Literature review

### 2.1 Conceptual framework of the study

**Fig. 1:**
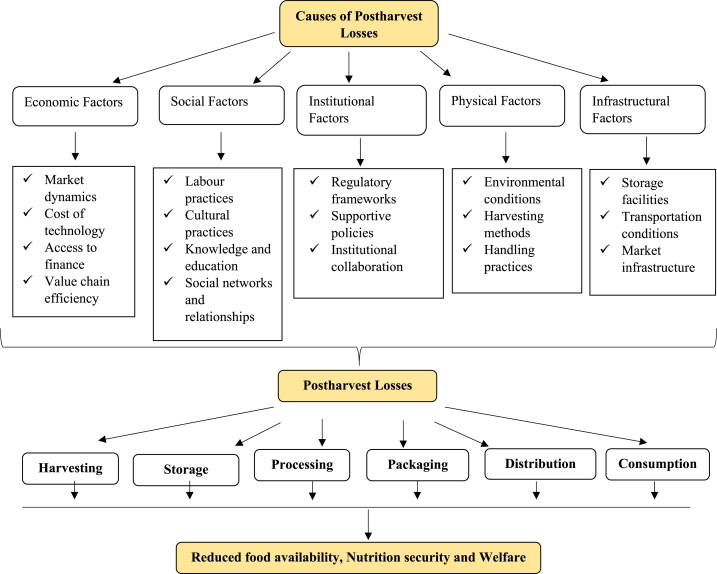
Conceptual framework showing the critical causal factors of postharvest losses in the vegetable supply chain.

The diagram in Fig. 1 illustrates the main causes of postharvest losses in the vegetable supply chain. It indicates that changes in weather, such as temperature, precipitation, and humidity, directly impact the quality of vegetables at every stage, from growth and harvest to postharvest handling [30]. For example, extreme temperatures or sudden fluctuations can harm vegetables. Moreover, high temperatures can speed up the deterioration of perishable produce, leading to rapid spoilage and increased losses [26]. Similarly, changes in humidity levels can affect the moisture content of vegetables, potentially causing them to rot [2]. Furthermore, climate change exacerbates these challenges by disrupting long-term weather patterns and increasing the frequency and intensity of extreme weather events [27,28]. This can disrupt normal growing seasons, affect crop yields and quality, and create favourable conditions for pests and diseases. For instance, prolonged droughts or heavy rainfall can damage crops, while warmer temperatures may encourage the spread of certain pests and pathogens, leading to further postharvest losses [31].

Economic factors are dominant in influencing postharvest losses within the vegetable supply chain. Market dynamics, encompassing fluctuations in demand and prices, can precipitate overproduction or undersupply, leading to surpluses or shortages of vegetables and ensuing losses [12]. Moreover, the accessibility and affordability of postharvest technologies are crucial; inflated costs associated with their adoption may dissuade investment, hindering efforts to mitigate losses [25]. Concurrently, limited credit or investment capital access can impede farmers' ability to implement improved postharvest practices. Additionally, inefficiencies within the supply chain, such as transportation delays or suboptimal handling practices, further exacerbate losses by prolonging transit times and compromising product quality [22]. Addressing these economic challenges requires interventions that enhance market stability, improve access to finance, and streamline supply chain operations to minimize postharvest losses effectively.

Social factors are pivotal in shaping postharvest losses within the vegetable supply chain, encompassing labour practices, cultural norms, knowledge dissemination, and social networks [4]. Inadequate availability of skilled labour for harvesting and handling can lead to mishandling, bruising, or damage to vegetables, thereby exacerbating losses [8]. Additionally, cultural beliefs or practices may influence postharvest handling techniques, potentially impacting product quality and longevity [33]. Moreover, a lack of awareness or training on proper postharvest practices can accelerate spoilage and waste [11]. However, strong social networks and collaborations among stakeholder farmers offer knowledge-sharing and resource-pooling opportunities, fostering collective action and innovative solutions to reduce losses [29]. By leveraging social capital and fostering collaboration, stakeholders can effectively address postharvest challenges and enhance the efficiency and sustainability of the vegetable supply chain.

Institutional factors exert considerable influence over postharvest losses within the vegetable supply chain, comprising regulatory frameworks, supportive policies, and institutional collaboration [8]. Insufficient regulations concerning food safety or quality standards can erect barriers to adopting best practices, thereby worsening postharvest losses [33]. Conversely, supportive policies and stakeholder collaborative efforts are pivotal in effectively addressing postharvest challenges. Supportive policies may include incentives for adopting innovative technologies, subsidies for infrastructure development, or grants for training programs to enhance postharvest practices [42]. Moreover, institutional collaboration fosters knowledge sharing, resource mobilization, and coordinated action, enabling stakeholders to implement holistic solutions that mitigate losses and enhance the resilience of the vegetable supply chain [11]. By cultivating an enabling institutional environment, policymakers and stakeholders can play a pivotal role in minimizing postharvest losses and promoting sustainable agricultural practices.

Physical factors are crucial determinants of postharvest losses within the vegetable supply chain, encompassing temperature and humidity control, packaging materials, storage facilities, and harvesting methods [26]. Moreover, effectively controlling temperature and humidity levels during storage and transportation is essential for preserving vegetable quality and minimizing spoilage [2]. Inadequate control in these aspects can accelerate deterioration, leading to significant losses. Similarly, choosing packaging materials and designs is critical in protecting vegetables from damage or contamination during handling and transit [13]. Poor packaging materials or designs can compromise the integrity of the produce, rendering it vulnerable to spoilage and deterioration. Furthermore, the availability and condition of storage facilities significantly impact postharvest losses [8]. Insufficient or improper storage facilities may lack adequate ventilation or temperature control, worsening losses due to accelerated spoilage. Additionally, improper harvesting techniques, such as rough handling or untimely harvesting, can cause physical damage or bruising to vegetables, reducing their shelf life and market value [32]. Therefore, addressing these physical factors is essential for minimizing postharvest losses and ensuring the quality and safety of vegetables throughout the supply chain.

Infrastructural factors significantly contribute to postharvest losses within the vegetable supply chain, encompassing transportation and market infrastructure, processing facilities, and packaging design [21]. Lack of transportation infrastructure or market integration can result in delays, temperature fluctuations, and increased losses during transit. For instance, inefficient cold chain logistics or inadequate storage facilities can lead to spoilage and deterioration of vegetables, particularly perishable varieties. Similarly, poor processing facilities or packaging designs may compromise product quality and marketability, increasing the risk of rejection or reducing consumer appeal [11]. Addressing these multifaceted factors necessitates a comprehensive approach involving stakeholder collaboration, investment in infrastructure and technology, supportive policies, and knowledge dissemination initiatives [23]. By enhancing transportation networks, upgrading processing facilities, improving packaging standards, and fostering market integration, stakeholders can mitigate postharvest losses, optimize resource utilization, and enhance the overall efficiency and sustainability of the vegetable supply chain.

## 3 Methodology

### 3.1 Description of the study area

**Fig. 2:**
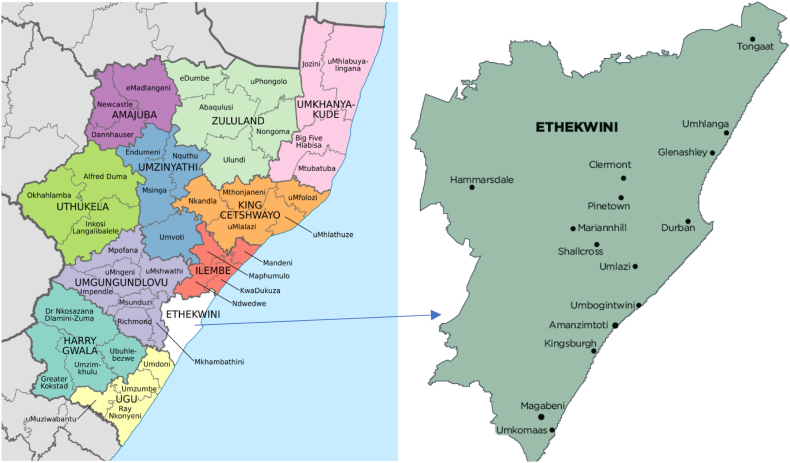
Map showing eThekwini Municipality.

**Source:** Cooperative Governance and Traditional Affairs (Cogta) (2020)

Fig. 2 displays the map of KwaZulu Natal and further zooms in the eThekwini Municipality. The eThekwini Municipality, characterized by a unique blend of urban and rural landscapes and abundant natural resources, presents a promising opportunity for leveraging the agricultural sector as a key driver for poverty alleviation. Establishing linkages between primary, secondary, and tertiary sectors, such as agribusiness and agro-processing, can catalyze regional economic growth and empowerment. However, the agricultural sector in the province faces significant hurdles, including economic challenges, inadequate infrastructure, and slow progress in land reform and redistribution efforts, as underscored by assessments from the eThekwini Municipality. There is a pressing need for improvements in government initiatives and agricultural policies to overcome these obstacles, essential for fostering sustainable agricultural development and poverty reduction.

Despite the challenges, the agricultural sector in KwaZulu-Natal benefits from favourable natural conditions, including reliable rainfall and fertile soils, contributing to its reputation for high productivity and specialized expertise across various farming disciplines. With approximately 6.5 million hectares of farming land, of which 82 % is suitable for extensive livestock production, and 18 % is designated as arable land, the province holds significant potential for agricultural advancement. Agriculture is prioritized in the Integrated Development Plans of municipalities, offering opportunities for economic development through surplus food and fiber production, job creation, and implementing agricultural programs within the eThekwini Metro, as outlined in the eThekwini Agribusiness Master Plan of 2022. By capitalizing on these strengths and addressing the identified challenges, KwaZulu-Natal can unlock the full potential of its agricultural sector, contributing to broader socioeconomic development and poverty alleviation efforts in the region. Fig. 2 zooms in the map of KwaZulu Natal and specifically eThekwini Municipality.

### 3.2 Research design

A mixed-methods approach was employed, integrating qualitative and quantitative techniques to explore variable relationships and forecast outcomes. This approach provided a comprehensive understanding of the subject. A cross-sectional research design was chosen for its cost-effectiveness and time efficiency, capturing data at a specific point in time without requiring longitudinal data collection. Descriptive analysis summarized and interpreted the data, while inferential analysis facilitated hypothesis testing and generalization of results to the broader population. Regression analysis evaluated the critical causal factors of postharvest losses in the vegetable supply chain, identifying significant predictors and their effects. These combined methodological approaches ensured a rigorous and thorough investigation of the research questions.

### 3.3 Sampling procedure, frame, and sample size

The study was conducted within the eThekwini Municipality, which was purposefully selected due to its high concentration of smallholder farmers engaged in vegetable farming activities. To ensure representative sampling, a multi-stratified random sampling approach was adopted. First, district municipalities were selected, followed by wards and villages with prevalent and operational agricultural activities. Within these villages, farmers were stratified based on the types of crops they produced, with a specific focus on vegetable farmers. The final stage involved the random selection of smallholder vegetable farmers as the unit of analysis for the study.

Cochran's formula was used to determine the sample size, considering a 95 % confidence level with a ±5 % precision. Assuming maximum variability (p = 0.5), the formula initially yielded a sample size (n_0_) 384. However, after a thorough data-cleaning process to rectify inconsistencies, inaccuracies, and missing values, 238 completed questionnaires were deemed suitable for analysis. This meticulous data cleaning was crucial for ensuring the dataset's reliability and integrity, enhancing the research findings' quality and accuracy. Consequently, the final sample size for analysis was n_0_ = 238, reflecting the rigorous approach taken to maintain the validity of the research outcomes.

### 3.4 Data collection

The study utilized a structured approach for data collection, employing local enumerators fluent in IsiZulu to ensure effective communication with participants. Extensive training was provided to these enumerators, who were familiarized with the questionnaire initially developed in English and then translated into IsiZulu. This preparation aimed to facilitate clear communication and enhance response accuracy. Before the primary data collection, a pilot test was conducted with 10 % of the respondents from Inchanga, an area outside the central study region. This test validated the questionnaire's consistency, reliability, and relevance and helped identify overlooked variables and address potential translation issues. The primary data collection involved 238 farmers, selected through a multi-stratified random sampling method within the eThekwini Municipality, known for its high concentration of smallholder vegetable farmers. The sampling frame was created by stratifying farmers according to the types of vegetables they grew, ensuring a representative sample of the broader population of smallholder farmers in the region. After administering the questionnaires, the enumerators checked the responses for completeness and accuracy before entering the data into the system for analysis. All procedures adhered to ethical guidelines and received prior approval from the Human and Social Sciences Research Ethics Committee (HSSREC) at the University of KwaZulu-Natal (Reference No.: HSSREC/00005449/2023).

### 3.5 Data analysis

The study thoroughly analyzed the collected data using descriptive and inferential statistics and the log-linear regression model. Initially, the data was captured, coded, and cleaned using Microsoft Excel, with outliers and incomplete questionnaires excluded. The cleaned dataset was then exported to Stata 18 for further analysis. Descriptive statistics were used to summarize the study population's characteristics through measures such as the mean and standard deviation, simplifying the data and clarifying the findings. Inferential statistics allowed researchers to make broader inferences and predictions about the entire population based on the sample data, extending the findings beyond the specific sample studied. As part of the inferential statistics approach, the log-linear regression model facilitated examining relationships between categorical variables, offering more profound insights into the data. These statistical techniques provided a robust framework for analysing the data and effectively addressing the research objectives.

### 3.6 Analytical tool

The study used log-linear regression analysis to investigate the critical factors contributing to postharvest losses in the vegetable supply chain. This method was chosen because it effectively examines relationships between variables with multiplicative properties, which is common in agricultural studies. Log-linear regression involves transforming variables using natural logarithms to linearize the relationships, making them suitable for regression analysis [5]. This transformation helps handle multiplicative relationships, capturing the proportional changes in postharvest losses related to changes in independent variables. This approach provides valuable insights for developing mitigation strategies.

### 3.7 Key advantages of log-linear regression include

• Modeling multiplicative relationships are often seen in agricultural systems.
• Ensuring unbiased parameter estimates by meeting specific assumptions, such as the linearity of the logarithmic relationship, independence of errors, homoscedasticity, normality of errors, absence of multicollinearity, and absence of perfect collinearity.

These features make log-linear regression a robust and valid tool for understanding postharvest losses. It is more suitable for analysing agricultural data than linear regression, which assumes additive relationships [24]. Using log-linear regression, the study provides a rigorous analytical framework tailored to the complexities of postharvest loss dynamics, enhancing the reliability and relevance of its findings for agricultural stakeholders.

Equation (1) below presents the general equation for log-linear regression:

(1) $$\log\left(Y_{i}\right)=\beta_{1}\;\log\left(X_{1i}\right)+\beta_{2}\;\log\left(X_{2i}\right)+\ldots +\beta_{k}\;\log\left(X_{ki}\right)+\varepsilon_{i}$$

Where:

Y_i_ = Dependent Variable (Postharvest losses)

β = Estimated parameters (Coefficients)

Log (X_k_) = Independent variables (e.g., Age, Experience, Market Access etc.)

ɛ = Error term.

This equation signifies that the dependent variable, postharvest losses (Y), is modelled as a function of the independent variables, with each variable's logarithmically transformed values serving as predictors. The coefficients (*β*_1_,*β*_2_ …,*β*_*k*_) represent the proportional change in postharvest losses for a one-unit change in the corresponding independent variable, while other variables remain constant.

### 3.8 Informed consent

Verbal informed consent was secured from all human participants involved in this study. Detailed explanations were provided concerning the study's objectives, potential advantages and risks, and participants' rights. The consent procedures received prior approval from the Human and Social Sciences Research Ethics Committee (HSSREC) at the University of KwaZulu-Natal (Reference No.: HSSREC/00005449/2023). Before their inclusion in the study, written consent was obtained from each participant. They were guaranteed confidentiality and informed of their prerogative to withdraw from the study at any point without facing any repercussions.

## 4 Results and discussions

This section primarily focuses on analyzing the acquired findings. First, it delves into the demographic attributes of smallholder vegetable farmers in the eThekwini Municipality. Subsequently, it examines the factors influencing postharvest losses within this demographic group. Moreover, it explores the empirical outcomes derived from the log-linear model utilized to assess the critical causal factors of postharvest losses within the vegetable supply chain.

### 4.1 Demographic characteristics of smallholder vegetable farmers in eThekwini municipality

The study conducted in eThekwini Municipality provided valuable insights into smallholder vegetable farmers' demographics and socio-economic dynamics. Table 1 presents the demographic and socioeconomic characteristics of these farmers. The average age of the farmers is 45 years, indicating a relatively middle-aged demographic compared to the national average. This suggests a potential openness to innovation and investment in essential farming skills. Although the average farmer has 12 years of formal education, which aligns with South Africa's secondary school completion, this level of educational attainment is still relatively low, hindering the adoption of new agricultural practices such as climate-smart agriculture [16]. With an average household size of six members and a monthly income of ZAR 8568.37, farming is a significant source of income for these households, although notable income disparities exist [18].

**Table 1:** The demographic and socioeconomic characteristics of smallholder farmers in eThekwini Municipality.

Variable description	Mean (n = 238)	Standard deviation
Age	45.11	9.54
Years in school	11.66	2.85
Household size	5.96	2.00
Total income	8568.37	6004.39
**Categorical Variables**	**Frequency**	**Percentage**

**Gender**		
Male	104	43.7 %
Female	134	56.3 %
**Marital Status**		
Single	184	77.31 %
Married	45	18.91 %
Widowed	7	2.94 %
Divorced	2	0.84 %
**Employment Status**		
Unemployed	180	75.63 %
Employed	41	17.23 %
Pension	13	5.46 %
Other	4	1.68 %
**Income source**		
Farming	186	78.15 %
Salary	21	8.82 %
Pension	16	6.72 %
Grant	9	3.78 %
Remittance	2	0.84 %
Other	4	1.68 %

Female farmers constitute 56 % of the demographic, underscoring their pivotal agricultural role. However, female-headed households face elevated risks of poverty and food insecurity, particularly in rural areas [9]. Marital status also influences farm dynamics, with 77 % of unmarried farmers potentially affecting operational effectiveness [7]. Additionally, a substantial proportion of farmers (76 %) are unemployed, highlighting their dependency on farming as their primary livelihood source [35]. The study emphasizes the importance of tailored interventions to address challenges related to education, income generation, gender disparities, and market engagement. These interventions are essential for enhancing the region's sustainability and profitability of smallholder vegetable farming.

### 4.2 Contributing Causes to postharvest losses among smallholder vegetable farmers in eThekwini municipality

**Fig. 3:**
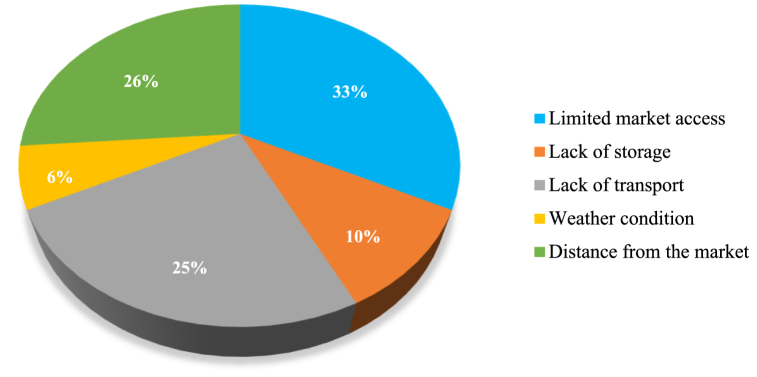
Contributing Causes to Postharvest Losses among Smallholder Vegetable Farmers in eThekwini Municipality.

Source: Field survey, 2023.

Fig. 3 illustrates the key factors contributing to postharvest losses experienced by smallholder vegetable farmers. The most prevalent issue, affecting 33 % of the farmers surveyed, is limited market access, suggesting significant challenges in reaching potential buyers, leading to surplus crops going unsold and wasted. About one-third of farmers face difficulties accessing suitable markets [20]. Additionally, 10 % of farmers reported a lack of storage facilities, indicating inadequate infrastructure for preserving harvested crops, leading to spoilage due to insufficient ventilation or temperature control [8]. About 25 % of respondents identified a lack of transport, highlighting difficulties in efficiently transporting crops to markets or storage facilities, resulting in delays and increased losses, especially during transit. Furthermore, 6 % of farmers cited adverse weather conditions, such as storms, droughts, or excessive rainfall, contributing to postharvest losses, impacting crop quality at every stage from growth to postharvest handling [30]. Lastly, 26 % of respondents mentioned the distance from the market as a challenge, with farmers farther away facing increased logistical difficulties and higher transportation costs, exacerbating postharvest losses. These findings underscore farmers' complex challenges in mitigating postharvest losses and highlight the need for targeted interventions to improve market access, infrastructure, transportation, and resilience to weather variability.

### 4.3 Critical causal factors of postharvest losses in the vegetable supply chain in eThekwini metropolitan

This section uses log-linear regression analysis to examine the critical causal factors of postharvest losses in the vegetable supply chain. Table 2 presents the estimates from the log-linear regression model based on a dataset of 238 observations. The analysis indicates a statistically significant fit for predicting postharvest losses. The likelihood ratio chi-square test produced a chi^2^ value of 64.82 with 16 degrees of freedom, resulting in a p-value of 0.00466. This allows us to reject the null hypothesis, suggesting that the independent variables in the model collectively explain the variability in postharvest losses. The log-likelihood value of −264.44297 indicates a good fit for the observed data. In contrast, the Pseudo R^2^ value of 0.711 implies that the model's independent variables explain approximately 71.1 % of the variability in postharvest losses. These results demonstrate the model's strong explanatory power in understanding the factors contributing to postharvest losses.

**Table 2:** Log-linear Regression Analysis of Critical Causal Factors Influencing Postharvest Losses in the Vegetable Supply Chain in eThekwini Municipality.

Variables	Coefficient	Std. error	P > z	[95 % conf. interval]	
Age	0.004	0.010	0.018^b^	−0.016249	0.023595
Years In School	0.018	0.033	0.050^a^	−0.046536	0.082027
Farm Experience	−0.090	0.027	0.036^a^	−0.043557	0.061643
Market Access	−0.927	1.681	0.051^a^	−2.367157	4.220857
Distance from the Market	0.040	0.079	0.016^b^	−0.114860	0.193866
Weather Conditions	−0.906	1.544	0.054^a^	−3.932051	2.120898
Lack of transport	−1.228	1.877	0.013^b^	−4.906756	2.450883
_cons	−0.958	0.950	0.313	−2.820058	0.903505

Log-linear regression.

Number of observations = 238.

LR chi^2^(16) = 64.82.

Prob > chi^2^ = 0.00466.

Log likelihood = −264.44297.

Pseudo R^2^ = 0.711.

a p < 0.05.

b p < 0.01.

Overall, the log-linear regression model provides valuable insights into the determinants of postharvest losses, identifying key areas for potential interventions or improvements in postharvest management practices to reduce losses and enhance agricultural productivity. Table 2 presents the significant critical causal factors within the vegetable supply chain as estimated by the model.

Age demonstrates a strong positive coefficient at the highly significant 1 % level, indicating a substantial positive relationship between age and postharvest losses within the vegetable supply chain. The results suggest that for every one-year increase in age among smallholder vegetable farmers, there is a 4 % increase in postharvest losses. Older farmers often face challenges in adopting modern postharvest management practices, leading to higher crop spoilage or waste levels [6]. Moreover, age influences farmers' capacity, knowledge, access to resources, and adoption of technologies, all significantly impacting postharvest handling and storage losses [19]. Given the substantial evidence supporting the role of age in postharvest loss dynamics, targeted support and training programs addressing age-related factors could be instrumental in mitigating postharvest losses and enhancing the efficiency of the vegetable supply chain in eThekwini Municipality. By equipping older farmers with the necessary knowledge and skills, interventions focused on improving postharvest practices can reduce losses along the supply chain, ultimately improving the profitability and sustainability of vegetable farming operations [8].

Years in school significantly predict postharvest losses among smallholder vegetable farmers, demonstrating a positive relationship and statistical significance at the 5 % level. This indicates that farmers with higher levels of education are more likely to adopt and implement effective postharvest management strategies, potentially reducing crop waste by 18 %. Education and training initiatives are crucial in enhancing farmers' knowledge and skills in postharvest handling and storage [4]. Such initiatives contribute significantly to mitigating postharvest losses and strengthening the economic viability of vegetable farming. Enhanced postharvest practices also improve the overall quality and marketability of produce, benefiting a wide range of stakeholders within the vegetable supply chain, including farmers, distributors, and consumers [36].

Farm experience reveals a negative relationship with postharvest losses and is statistically significant at the 5 % level. This implies that increased farming experience correlates with a 9 % reduction in postharvest losses among smallholder vegetable farmers. The crucial role of accumulated knowledge and practical skills in reducing losses within the vegetable supply chain [20,28]. Experienced farmers are more adept at effectively harvesting, handling, and storage practices, leading to significant waste reductions [8]. This has important implications for the vegetable supply chain, including improved profitability, sustainability, quality assurance, and the potential for enhanced livelihoods and economic viability for smallholder farmers.

The impact of distance to the market on postharvest losses has significant implications for the entire vegetable supply chain. The positive coefficient and statistical significance at the 1 % level indicate that for every kilometer increase in distance to the market, postharvest losses increase by 4 %. Increased distance to markets correlates with higher postharvest losses [41]. This relationship suggests that logistical challenges associated with transportation, including prolonged travel times and inadequate infrastructure, contribute to increased spoilage and wastage of vegetables [11]. Postharvest losses in the vegetable supply chain affect farmers' income and have ripple effects on other stakeholders, including distributors, retailers, and consumers [36]. As postharvest losses increase with distance to the market, distributors and retailers may face challenges in sourcing fresh produce, leading to reduced availability and increased consumer prices. Additionally, food waste along the supply chain contributes to environmental degradation and exacerbates food insecurity [10].

The negative coefficient and statistical significance at the 1 % level for lack of transport in the vegetable supply chain highlight the substantial impact limited access to transportation services has on increasing postharvest losses among smallholder vegetable farmers by 22 %. Inadequate transportation infrastructure and services contribute to delays in delivering harvested vegetables to markets or processing facilities, resulting in increased spoilage and waste [9]. These losses undermine the economic viability of smallholder farmers and exacerbate food insecurity while impeding efforts to improve supply chain efficiency and resilience [13]. The implications extend throughout the vegetable supply chain, affecting distributors, retailers, and consumers by reducing the availability of fresh produce and potentially driving up prices.

The negative coefficient and statistical significance at the 5 % level for weather conditions in the vegetable supply chain suggest that adverse weather conditions significantly contribute to postharvest losses among smallholder vegetable farmers. Extreme weather events, such as droughts, floods, and storms, can harm agricultural production and postharvest management practices [4]. Additionally, adverse weather conditions directly impact crop yields, exacerbate logistical challenges, and reduce the effectiveness of postharvest management practices [14]. By addressing these challenges, stakeholders can minimize postharvest losses, enhance the resilience of smallholder vegetable farming systems, and ensure a more sustainable and secure vegetable supply chain.

Market access shows a negative coefficient and is statistically significant at the 5 % level, indicating that improved market access may lead to a 92 % reduction in postharvest losses among smallholder vegetable farmers. Market access plays a critical role in mitigating postharvest losses and enhancing the economic viability of smallholder farmers [36]. Moreover, improved market access facilitates the timely and efficient transportation of harvested vegetables to markets or processing facilities, reducing spoilage and waste [7]. Additionally, enhanced market access opens opportunities for farmers to explore diverse market channels, negotiate better prices, and adopt postharvest management practices that improve their produce's quality and shelf life [34]. Investing in infrastructure, such as roads, transportation networks, and market linkages, can help overcome barriers to market access, unlocking the full potential of smallholder vegetable farming and contributing to rural communities' economic empowerment and food security.

### 4.4 Practical limitations and further research areas

Firstly, the research is based on data collected from 238 smallholder vegetable farmers in eThekwini Municipality. Although this provides valuable insights, the findings may not be generalizable to other regions or larger populations. Future studies could expand the sample size and include multiple regions for broader applicability. Additionally, the data used were self-reported by farmers, which may be subject to recall bias or inaccuracies. Despite efforts to verify the data, future research could incorporate more objective measures, such as field observations and third-party audits, to enhance data accuracy.

The study's cross-sectional nature captures data at a single point, limiting the ability to assess changes over time. Longitudinal studies are needed to understand the dynamics of postharvest losses and the long-term effects of interventions. Furthermore, our focus on smallholder vegetable farmers may not capture the experiences and challenges of larger commercial farms. Future research could compare postharvest losses across different farm sizes and types to identify specific needs and solutions.

Looking ahead, several potential areas for further research have been identified. Conducting longitudinal studies to track changes in postharvest losses over time and the long-term impact of various interventions would provide deeper insights into the effectiveness and sustainability of strategies to reduce losses. Expanding the research to include multiple regions or countries to compare postharvest losses and identify region-specific factors and solutions could help tailor interventions to different agricultural contexts. Investigating the impact of technological innovations, such as improved storage facilities, transportation technologies, and digital market access platforms, on reducing postharvest losses and assessing the adoption and effectiveness of these technologies could inform policy and practice.

Exploring how climate change affects postharvest losses and the effectiveness of adaptation strategies is another crucial area for future research. This could focus on developing and promoting climate-resilient postharvest practices and technologies. Additionally, conducting detailed economic impact analyses to quantify the financial losses associated with postharvest waste and the potential economic benefits of reducing these losses could help policymakers and stakeholders make informed investment decisions. Examining the gender and social dynamics of postharvest losses, particularly the roles and challenges faced by female farmers and marginalized groups, and identifying targeted interventions to support these groups could enhance agricultural productivity and food security.

## 5 Conclusion and recommendations

The study aimed to investigate the critical causal factors contributing to postharvest losses (PHLs) along the vegetable supply chain. Using structured questionnaires, a cross-sectional research design and a multi-stratified random sampling technique were employed to collect primary data from 238 farmers. The study used both descriptive statistics and log-linear regression to analyze the data. The study results revealed that the majority (56 %) of the farmers were females with an average age of 45 years and a household size of 5 people per household. Farmers spent an average of 12 years in school with the majority being unemployed (76 %), relying on farming for income (78 %). Moreover, 33 % of the farmers indicated that limited market access contributed to the postharvest losses, while other farmers highlighted that weather conditions (6 %), lack of transport (25 %), distance to the market (26 %) and lack of storage (10 %) also contribute to their PHLs. Moreover, the study's findings shed light on key factors influencing postharvest losses within the vegetable supply chain in the eThekwini Municipality. Age, years of schooling, and farm experience all play significant roles in determining the extent of postharvest losses among smallholder vegetable farmers. Older farmers may struggle to adopt modern postharvest management practices, while higher education and training are associated with decreased losses. Bridging the gap between traditional and contemporary farming practices through targeted support and education initiatives is crucial for mitigating losses and improving the efficiency of the supply chain. Moreover, challenges related to distance to the market and lack of transport infrastructure significantly impact postharvest losses, underscoring the importance of investing in transportation infrastructure to facilitate efficient produce transportation. Additionally, improved market access can reduce postharvest losses by enabling farmers to sell their produce more efficiently. Therefore, addressing these factors through targeted interventions and investments can contribute to reducing postharvest losses and improving the economic viability and sustainability of vegetable farming operations and the vegetable supply chain.

The study recommends that.

o **Supportive Policy Environment**: Create an enabling policy environment that supports the adoption of innovative postharvest management technologies and practices. This includes providing financial incentives, subsidies, and technical assistance to farmers for investing in postharvest infrastructure, such as storage facilities and packaging materials, and adopting best practices for minimizing losses along the supply chain. Additionally, streamline regulatory processes and remove barriers to market access for smallholder farmers to encourage entrepreneurship and investment in the vegetable sector.
o **Infrastructure Development**: Develop and improve transportation infrastructure, including roads, cold chain facilities, and storage facilities, to facilitate the efficient and timely movement of produce from farms to marketplaces or storage facilities. Government policies should prioritize investments in infrastructure projects that benefit rural areas and improve market access for smallholder farmers.
o **Promote Market Access**: Implement policies to promote market access for smallholder vegetable farmers, including initiatives to improve market linkages, establish market information systems, and support the development of local markets. Additionally, incentivize private sector involvement in building market infrastructure and facilitating market access for smallholder farmers.
o **Climate-Resilient Farming Practices**: Develop and promote climate-resilient farming practices and technologies that help mitigate the impact of adverse weather conditions on crop yields and postharvest losses. Government policies should incentivize the adoption of sustainable farming practices, such as agroforestry, soil conservation, and water management techniques, to enhance the resilience of vegetable farming systems to climate change.
o **Age-Responsive Training Programs:** Implement training programs tailored to the needs of farmers of different ages, particularly focusing on older farmers. These programs should offer guidance on modern postharvest management techniques and technologies, considering the challenges older farmers may face in adopting new practices. By providing age-responsive training, we can ensure that farmers of all ages have the knowledge and skills necessary to minimize postharvest losses effectively, ultimately enhancing the efficiency and sustainability of agricultural practices.

## CRediT authorship contribution statement

**Siphesihle Qange:** Writing – review & editing, Writing – original draft, Resources, Project administration, Methodology, Investigation, Funding acquisition, Formal analysis, Data curation, Conceptualization. **Lelethu Mdoda:** Supervision. **Asanda Mditshwa:** Supervision.

## Ethical clearance

Ethical clearance was obtained from the Human and Social Sciences Research Ethics Committee (HSSREC) of the University of KwaZulu-Natal (Reference No.: HSSREC/00005449/2023).

## Data availability statement

The dataset used in this study is available upon request from the corresponding author. Access to the data is subject to approval from the relevant institutional review boards and compliance with data protection regulations. Requests for data access should be directed to [corresponding author's email address]. Access to the dataset will be granted following a formal data-sharing agreement to ensure adherence to ethical and legal requirements governing data use and confidentiality. This approach safeguards study participants' privacy and confidentiality while facilitating the reproducibility and transparency of the research findings.

## Consent for publication

I, Siphesihle Qange, hereby grant permission for the publication of this study, “Evaluating the Critical Causal Factors of Postharvest Losses in the Vegetable Supply Chain in eThekwini Metropolitan: The Log-linear regression model.”

I understand that the material to be published may include personal information, and I consent to its inclusion. I understand that efforts will be made to ensure my anonymity and confidentiality and that my personal information will not be disclosed without my explicit consent.

I understand that the publication may be disseminated in various formats, including print, electronic, and online platforms, and that it may be accessed by individuals worldwide. I acknowledge that I have been provided with an opportunity to review the material to be published and to request any necessary revisions or modifications.

I confirm that I have read and understood the terms of this consent form, and I voluntarily agree to its provisions. I understand that my participation in the research study is entirely voluntary, and that I may withdraw my consent for publication at any time without penalty.

## Funding

The 10.13039/501100001321National Research Foundation has funded the study. Reference no.: MND210418595675.

## Declaration of competing interest

The authors declare that they have no known competing financial interests or personal relationships that could have appeared to influence the work reported in this paper.
